# Low-temperature redetermination of 3,4,5,6-tetra­hydro­pyrimidin-2(1*H*)-one

**DOI:** 10.1107/S160053680801115X

**Published:** 2008-04-26

**Authors:** Mohd. Razali Rizal, Isha Azizul, Seik Weng Ng

**Affiliations:** a Department of Chemistry, University of Malaya, 50603 Kuala Lumpur, Malaysia

## Abstract

The low-temperature structure of the title compound, C_4_H_8_N_2_O, is ordered, whereas the central methyl­ene groups is disordered in the reported room-temperature structure. The molecule lies across a mirror plane; adjacent mol­ecules are linked by an N—H⋯O hydrogen bond into a chain.

## Related literature

For the room-temperature, disordered structure of tetra­hydro­pyrimidin-2(1*H*)-one, see: Calogero *et al.* (1980 ▶).
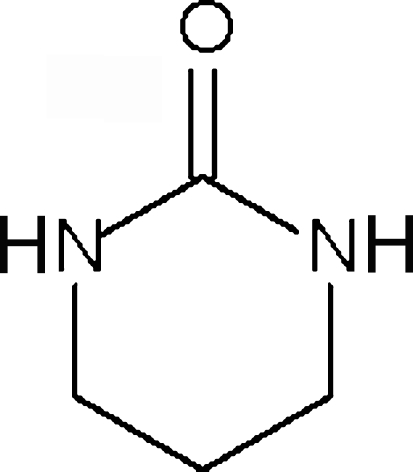


## Experimental

### 

#### Crystal data

C_4_H_8_N_2_O
*M* *_r_* = 100.12
Orthorhombic, 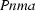

*a* = 9.9958 (1) Å
*b* = 7.1327 (1) Å
*c* = 6.7365 (1) Å
*V* = 480.29 (1) Å^3^
*Z* = 4
Mo *K*α radiation
μ = 0.10 mm^−1^
*T* = 100 (2) K
0.35 × 0.20 × 0.15 mm

#### Data collection

Bruker SMART APEX diffractometer
Absorption correction: none
6595 measured reflections
749 independent reflections
719 reflections with *I* > 2σ(*I*)
*R*_int_ = 0.020

#### Refinement

*R*[*F* ^2^ > 2σ(*F* ^2^)] = 0.036
*wR*(*F* ^2^) = 0.110
*S* = 1.06
749 reflections
55 parameters
All H-atom parameters refined
Δρ_max_ = 0.49 e Å^−3^
Δρ_min_ = −0.21 e Å^−3^

### 

Data collection: *APEX2* (Bruker, 2007 ▶); cell refinement: *SAINT* (Bruker, 2007 ▶); data reduction: *SAINT*; program(s) used to solve structure: *SHELXS97* (Sheldrick, 2008 ▶); program(s) used to refine structure: *SHELXL97* (Sheldrick, 2008 ▶); molecular graphics: *X-SEED* (Barbour, 2001 ▶); software used to prepare material for publication: *publCIF* (Westrip, 2008 ▶).

## Supplementary Material

Crystal structure: contains datablocks global, I. DOI: 10.1107/S160053680801115X/bv2094sup1.cif

Structure factors: contains datablocks I. DOI: 10.1107/S160053680801115X/bv2094Isup2.hkl

Additional supplementary materials:  crystallographic information; 3D view; checkCIF report

## Figures and Tables

**Table 1:** Hydrogen-bond geometry (Å, °)

*D*—H⋯*A*	*D*—H	H⋯*A*	*D*⋯*A*	*D*—H⋯*A*
N1—H1⋯O1^i^	0.89 (1)	1.97 (1)	2.864 (1)	178 (1)

Symmetry code: (i) 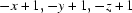.

**Fig. 1.**
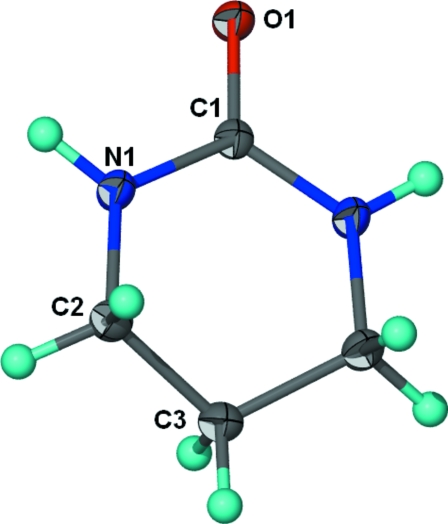
Thermal ellipsoid plot of tetrahydropyrimidin-2(1H)-one at the 70% probability level; hydrogen atoms are drawn as spheres of arbitrary radius.

## supplementary crystallographic information

### Comment

The crystal structure of tetrahydropyrimidin-2(1*H*)-one (Scheme I) was refined as a disorder model, the second of the three methylene carbon atoms being disordered about a mirror plane by a ratio of 0.7:0.3 (Calogero *et al.*, 1980). However, the structure is ordered at low temperature (Fig. 1); adjacent molecules are linked by a N–H···O hydrogen bond into a chain motif.

### Experimental

The commercially available compound was crystalline. A large block was cut into a smaller specimen.

### Refinement

All hydrogen atoms were located in a difference Fourier map, and were freely refined.

### Crystal data

**Table:**

C_4_H_8_N_2_O	*F*_000_ = 216
*M**_r_* = 100.12	*D*_x_ = 1.385 Mg m^−^^3^
Orthorhombic, *P**n**m**a*	Mo *K*α radiation λ = 0.71073 Å
Hall symbol: -P 2ac 2n	Cell parameters from 4972 reflections
*a* = 9.9958 (1) Å	θ = 3.0–30.4º
*b* = 7.1327 (1) Å	µ = 0.10 mm^−^^1^
*c* = 6.7365 (1) Å	*T* = 100 (2) K
*V* = 480.29 (1) Å^3^	Block, colorless
*Z* = 4	0.35 × 0.20 × 0.15 mm

### Data collection

**Table:**

Bruker SMART APEXII diffractometer	719 reflections with *I* > 2σ(*I*)
Radiation source: fine-focus sealed tube	*R*_int_ = 0.020
Monochromator: graphite	θ_max_ = 30.0º
*T* = 100(2) K	θ_min_ = 3.7º
φ and ω scans	*h* = −12→13
Absorption correction: none	*k* = −9→10
6595 measured reflections	*l* = −9→9
749 independent reflections	

### Refinement

**Table:**

Refinement on *F*^2^	Secondary atom site location: difference Fourier map
Least-squares matrix: full	Hydrogen site location: inferred from neighbouring sites
*R*[*F*^2^ > 2σ(*F*^2^)] = 0.036	All H-atom parameters refined
*wR*(*F*^2^) = 0.110	*w* = 1/[σ^2^(*F*_o_^2^) + (0.0818*P*)^2^ + 0.084*P*] where *P* = (*F*_o_^2^ + 2*F*_c_^2^)/3
*S* = 1.06	(Δ/σ)_max_ = 0.001
749 reflections	Δρ_max_ = 0.49 e Å^−^^3^
55 parameters	Δρ_min_ = −0.21 e Å^−^^3^
Primary atom site location: structure-invariant direct methods	Extinction correction: none

### Fractional atomic coordinates and isotropic or equivalent isotropic displacement parameters (Å^2^)

**Table:**

	*x*	*y*	*z*	*U*_iso_*/*U*_eq_	
O1	0.48291 (8)	0.7500	0.51927 (11)	0.0143 (2)	
N1	0.58480 (7)	0.58713 (8)	0.27287 (9)	0.0140 (2)	
C1	0.54996 (10)	0.7500	0.36064 (14)	0.0113 (2)	
C2	0.67121 (7)	0.57605 (10)	0.09858 (11)	0.0145 (2)	
C3	0.65258 (11)	0.7500	−0.02874 (14)	0.0146 (3)	
H1	0.5642 (13)	0.4836 (18)	0.3405 (19)	0.023 (3)*	
H21	0.6483 (11)	0.4643 (17)	0.0232 (17)	0.017 (3)*	
H22	0.7668 (11)	0.5621 (17)	0.1397 (17)	0.017 (2)*	
H31	0.5609 (18)	0.7500	−0.089 (3)	0.023 (4)*	
H32	0.7163 (18)	0.7500	−0.140 (3)	0.023 (4)*	

### Atomic displacement parameters (Å^2^)

**Table:**

	*U*^11^	*U*^22^	*U*^33^	*U*^12^	*U*^13^	*U*^23^
O1	0.0176 (4)	0.0122 (4)	0.0132 (4)	0.000	0.0045 (3)	0.000
N1	0.0188 (4)	0.0099 (3)	0.0133 (3)	0.0003 (2)	0.0047 (2)	0.00012 (19)
C1	0.0109 (4)	0.0111 (5)	0.0118 (4)	0.000	−0.0008 (3)	0.000
C2	0.0173 (4)	0.0128 (4)	0.0133 (4)	0.0010 (2)	0.0039 (2)	−0.0008 (2)
C3	0.0177 (5)	0.0145 (5)	0.0117 (4)	0.000	0.0013 (3)	0.000

### Geometric parameters (Å, °)

**Table:**

O1—C1	1.2614 (12)	C2—H21	0.972 (12)
N1—C1	1.3492 (8)	C2—H22	1.000 (11)
N1—C2	1.4597 (9)	C3—C2^i^	1.5198 (9)
N1—H1	0.892 (13)	C3—H31	1.001 (18)
C1—N1^i^	1.3492 (8)	C3—H32	0.982 (19)
C2—C3	1.5198 (9)		
			
C1—N1—C2	123.49 (6)	N1—C2—H22	110.4 (7)
C1—N1—H1	115.4 (8)	C3—C2—H22	110.8 (7)
C2—N1—H1	120.2 (8)	H21—C2—H22	106.8 (10)
O1—C1—N1	120.56 (4)	C2^i^—C3—C2	109.45 (8)
O1—C1—N1^i^	120.56 (4)	C2^i^—C3—H31	109.9 (5)
N1—C1—N1^i^	118.86 (9)	C2—C3—H31	109.9 (5)
N1—C2—C3	109.71 (6)	C2^i^—C3—H32	110.5 (5)
N1—C2—H21	109.0 (7)	C2—C3—H32	110.5 (5)
C3—C2—H21	110.2 (7)	H31—C3—H32	106.7 (15)
			
C2—N1—C1—O1	−174.85 (8)	C1—N1—C2—C3	−30.95 (10)
C2—N1—C1—N1^i^	7.00 (14)	N1—C2—C3—C2^i^	52.71 (10)

Symmetry codes: (i) *x*, −*y*+3/2, *z*.

### Hydrogen-bond geometry (Å, °)

**Table:**

*D*—H···*A*	*D*—H	H···*A*	*D*···*A*	*D*—H···*A*
N1—H1···O1^ii^	0.89 (1)	1.97 (1)	2.864 (1)	178 (1)

Symmetry codes: (ii) −*x*+1, −*y*+1, −*z*+1.
